# Early Flowering as a Drought Escape Mechanism in Plants: How Can It Aid Wheat Production?

**DOI:** 10.3389/fpls.2017.01950

**Published:** 2017-11-17

**Authors:** Yuri Shavrukov, Akhylbek Kurishbayev, Satyvaldy Jatayev, Vladimir Shvidchenko, Lyudmila Zotova, Francois Koekemoer, Stephan de Groot, Kathleen Soole, Peter Langridge

**Affiliations:** ^1^School of Biological Sciences, Flinders University, Bedford Park, SA, Australia; ^2^School of Agriculture, Food and Wine, University of Adelaide, Urrbrae, SA, Australia; ^3^Faculty of Agronomy, S. Seifullin Kazakh AgroTechnical University, Astana, Kazakhstan; ^4^Sensako, Bethlehem, South Africa

**Keywords:** drought avoidance, drought escape, drought tolerance, early flowering time, early maturing, high yield potential, terminal drought, wheat

## Abstract

Drought escape (DE) is a classical adaptive mechanism which involves rapid plant development to enable the completion of the full life-cycle prior to a coming drought event. This strategy is widely used in populations of native plants, and is also applicable to cereal crops such as wheat. Early flowering time and a shorter vegetative phase can be very important for wheat production in conditions of terminal drought since this can minimize exposure to dehydration during the sensitive flowering and post-anthesis grain filling periods. A gradual shift toward early flowering has been observed over the last century of wheat breeding in countries with a Mediterranean-type climate and frequent terminal drought. This trend is predicted to continue for wheat production in the coming years in response to global climate warming. The advantage of early flowering wheat is apparent under conditions of impending terminal drought, and modern varieties are significantly more productive due to minimization of the risk associated with drought stress. Under favorable conditions, a short vegetative phase can result in reduced plant biomass due to the reduction in time available for photosynthetic production and seed nutrient accumulation. However, high yield potential has been reported for the development of both shallow and deep roots, representing plasticity in response to drought in combination with the early flowering trait. Wheat productivity can be high both in well-watered and drought-affected field trials, where an efficient strategy of DE was associated with quick growth, yield potential and water use efficiency. Therefore, early flowering provides a promising strategy for the production of advanced drought-adapted wheat cultivars.

## Preamble

The topic of early flowering as a DE mechanism is extremely important and has very long history of discussion amongst a wide range of plant biologists. This Mini-review is not designed to be comprehensive but instead is focused on a conceptual vision of the advantages and limitations of the topic based on published data. We discuss the topic of early flowering and DE from different angles, with high wheat production as a final target. We hope our paper can initiate further research and fruitful discussions.

## Comparison of Drought Escape and Drought Avoidance

Drought escape is one of many strategies employed by plants under conditions where water limitation late in the growing season is likely, and it ensures that the plants can complete their life-cycle quickly during the brief period of favorable conditions. Initially DE was described as a classical mechanism for ephemeral native plants, developed over a long period of evolution, mostly in perennial species such as bulbs and shrubs (Levitt, 1980; Turner, 1986; Ludlow, 1989; Loss and Siddique, 1994; Chaves et al., 2003; Zlatev and Lidon, 2012; Dolferus, 2014; Kooyers, 2015), but also in some annual grasses, for example in California vernal pools (Barry, 1995). DE or an ‘ephemeral strategy’ assumes a very high metabolic rate, resulting in progressive cell expansion and division. Open stomata and high gas exchange rates facilitate effective photosynthesis and photo-respiration with low WUE, and very rapid plant development (Bodner et al., 2015; Kooyers, 2015).

In contrast, DA can be defined as a mechanism of slow plant growth, associated with small or closed stomata with reduced photosynthesis as well as low cell metabolism in general. High WUE minimizes water loss and prepares the plants for the changing environment and coming drought. This is also commonly referred to as the ‘succulent strategy’ (Arraudeau, 1989; Ludlow, 1989; Slafer et al., 1994; Verslues et al., 2006; Chew and Halliday, 2011; Farooq et al., 2012; Fang and Xiong, 2015).

In reality, the classification of plants into the discrete categories of DE or DA is not entirely accurate since they take place at different times: prior to and after the occurrence of a drought event, respectively. This means that theoretically plants can use a combination of DE and DA or other strategies for drought tolerance.

Drought is a complex abiotic stress which can act at any stage of plant growth to varying degrees of severity. For the purposes of this mini-review we will identify three major periods of cereal plant development that can be affected by drought: (1) Vegetative (before transition to the reproductive stage); (2) Pre-anthesis (from tillering and booting until full flowering); and (3) Post-anthesis or terminal (after flowering and until maturity). The severity of drought can be determined by the actual water deficit experienced by plants, whereas the general categories of light, mild and severe levels of drought stress are usually used in publications.

Under favorable conditions, DE and the ‘ephemeral strategy’ dictate a short and fixed duration of the plant life-cycle. However, such plants still react in the sudden-onset of stress conditions. If mild drought occurs early in the vegetative stage of a DE plant’s development, it can cause a strong negative effect on growth, and such plants may be unable to survive unless they can quickly switch their response to a more effective mechanism of drought tolerance (Ludlow, 1989; Loss and Siddique, 1994; Chaves et al., 2003). However, under conditions of terminal drought, DE plants accelerate their growth in an attempt to complete their life-cycle and minimize their exposure to the abiotic stress. Plants from many different species can speed-up their post-anthesis development in conditions of terminal drought (Kottmann et al., 2016). This indicates that DE is a universal characteristic of development for plants close to maturity (Turner, 1986). This was made especially clear in studies with the model species *Arabidopsis thaliana*, where selection under drought conditions can favor early flowering (Meyre et al., 2001; Verslues and Juenger, 2011; Kenney et al., 2014).

Drought escape or the ‘succulent strategy’ can be helpful in both stages of plant development, if mild drought is relatively short or has occurred several times. Plants with DA react with slower metabolic processes, minimal transpirational loss, increased water uptake from deeper roots, higher WUE and leaf rolling (Arraudeau, 1989; Ludlow, 1989; Loss and Siddique, 1994; Slafer et al., 1994; Kadioglu and Terzi, 2007; Miyazawa et al., 2011; Kooyers, 2015). However, DA can also be fatal in a prolonged dry period regardless of plant developmental stage. Therefore, DE and DA represent alternative strategies that either speed-up or slow-down plant development, where the timing of drought occurrence (terminal, but not early to middle development) and drought duration (short, but not long) are the critical factors favoring the deployment of DE and DA, respectively.

## Mechanism of Drought Escape in Plants: Early Flowering

Despite the classical determination of DE in plant ecology as characteristic of ‘ephemeral native plants’, important cereals, such as wheat and barley, can also display a mechanism similar to DE, named ‘earliness’ or ‘early flowering’ (Dolferus, 2014).

Rapid development of plants is largely determined by the transition from the vegetative to reproductive stages, where the ‘time to initiation of flowering’ or more typically the ‘FT’ trait has a strong genetic background based on three groups of genes, vernalization (*Vrn*), photoperiod (*Ppd*) and earliness *per se* (*Eps*). Due to the very high importance of this trait for cereal production, the genetics of FT is well studied, with a long history of documented research (Distelfeld et al., 2009; Greenup et al., 2009; Chew and Halliday, 2011; Bentley et al., 2013; Riboni et al., 2013; Zheng et al., 2013; Campoli and Korff, 2014; Kamran et al., 2014; Khanna-Chopra and Singh, 2015). EFT results in the more rapid transition from vegetative to reproductive stages of cereal plant development, where tillering, spike development, flowering, and finally, EM follow a SVP. Such plants were also named ‘short-season’ or ‘short-cycle’ genotypes, and can effectively escape drought (Turner, 1986; Loss and Siddique, 1994; Acevedo et al., 1999; Chaves et al., 2003; Song et al., 2013). EFT and EM are complex traits determined by the interaction of several groups of genes from major pathways including response to vernalization, photoperiod, and gibberellic acid biosynthesis. The genes either promoting or antagonistic to these pathways are well studied (Distelfeld et al., 2009; Greenup et al., 2009; Chew and Halliday, 2011; Bentley et al., 2013; Riboni et al., 2013; Zheng et al., 2013; Campoli and Korff, 2014; Kamran et al., 2014; Khanna-Chopra and Singh, 2015), but this topic is outside the focus of the current paper.

Interestingly, reports of the genetic transformation of genes involved in drought tolerance are wide-spread in the literature, whereas there is no comparable data available regarding transgenic plants with modified EFT/EM traits (Lawlor, 2013).

There are reports providing evidence that evolution can favor EFT/EM traits in native populations of plants even without the pressure of oncoming drought stress. EFT plants of ornamental annual phlox, *Phlox drummondii*, produced a much greater number of offspring while the number of progeny produced by later flowering plants declined exponentially (Kelly and Levin, 2000). Phlox EFT plants produced more biomass compared to later flowering plants, which may indicate more active metabolism, photosynthesis, nutrient use and better growth of EFT plants with earlier completion of the life-cycle.

In regard to cereals, breeders can select genotypes with a FT most suitable for their environment. Cereals with EFT/EM traits can escape stressful conditions and complete their life-cycle faster, minimizing the duration of overlap between their growth and the oncoming stress – an important outcome for plant breeding and agricultural production (Stapper and Fischer, 1990; Dolferus, 2014).

## Drought Escape and Early Flowering Time/Early Maturing Can Provide Benefits to Plants Under Drought Stress

Under the current conditions of our changing climate, with a 1°C global temperature increase, wheat yield is projected to decline between 4.1 and 6.4% (Liu et al., 2016). This does not automatically mean that crops will experience greater drought, since the associated changes in precipitation are harder to model. Nevertheless, it is very likely that many regions will be more strongly affected by drought.

In such environments, natural selection favors EFT plants as more likely to be adapted to the approaching drought (Bodner et al., 2015; Kooyers, 2015). This was found to be true for the wild annual species *Avena barbata* (Sherrard and Maherali, 2006), and for 279 other species reviewed and documented by Parmesan and Yohe (2003), which predictably flowered earlier in a warmer environment, as well as for 234 spring accessions of *Arabidopsis thaliana* grown under drought (Kenney et al., 2014). However, in the same model plant species, EFT and DE showed benefits only in conditions of terminal drought, while repeated or continuous mild drought stress caused impaired growth and disadvantaged plant fitness (Loss and Siddique, 1994; Schmalenbach et al., 2014). Other plant species were reported to have different responses to early onset of drought, for example, one of two Mediterranean shrub species, *Erica multiflora*, showed EFT while another species, *Globularia alypum*, had no changes in time to flowering (Bernal et al., 2011). Strong changes in EFT were demonstrated experimentally in analyses of *Brassica rapa* hybrids, where micro-evolutionary changes for early flowering were fixed and inherited within only a few generations under drought (Franks et al., 2007; Franks, 2011).

Through artificial selection by breeders, plants with early flowering can limit their vegetative growth and enable reproductive growth to occur before the terminal stress, in a process that usually correlates with early maturity (Bodner et al., 2015). Nevertheless, some variability between FT and time for grain filling until maturity is reported (Turner, 1986; Farooq et al., 2014). Crops with EFT/EM traits can produce higher and more stable yields under drought conditions (Ludlow and Muchow, 1990; Fukai et al., 1998; Turner et al., 2001; Serraj et al., 2003; Khanna-Chopra and Singh, 2015). It can also result in the production of more seeds under water limitation in EFT/EM pearl millet and sorghum (Van Oosterom et al., 1996; Serraj et al., 2003; Reddy et al., 2009), *Canola juncea* (Bueckert and Clarke, 2013; Zhang H. et al., 2013), and groundnuts, *Arachis hypogaea* L., compared to cultivars with regular FT (Serraj et al., 2003; Clavel et al., 2005). Four cultivars of chickpea (*Cicer arieticum* L.) and seven mutant mungbean lines (*Vigna radiata* (L.) Wilczek) flowered 2–4 weeks earlier than traditional cultivars and parental forms, respectively, displaying enhanced seed yield (Serraj et al., 2003; Gaur et al., 2008) and up-to 30–50% in increase seed production overall (Malik et al., 1989). Conflicting conclusions exist about early flowering in rice. In Thailand, the yield of rice with a SVP was more stable over 17 years of analysis, where cultivars with 13 days EFT compared to the popular KDML105 could potentially reduce the risk of lower yield due to DE (Fukai et al., 1998). Supporting results were recently published with EFT/EM rice in Pakistan associated with increased yield under drought (Kumar et al., 2016). In contrast, in China, no significant correlations have been found between EFT/EM and relative grain yield in rice plants at the reproductive stage in a dry environment (Yue et al., 2006). Results of this nature led to the general assumption, still held by many, that EFT/EM is “not applicable in breeding for crops with high yields under drought conditions” (Krannich et al., 2015). However, this sceptical conclusion and others have asserted that the benefit of EFT/EM for crop production under drought stress should be subject to further questioning (Fischer, 1979; Turner, 1986). The nature of such a strategy has a firm biological basis, since a survival strategy will take precedence over production in native plants with DE (Ludlow, 1989).

## Early Flowering Time and Early Maturing in Wheat Represents Successful Strategies Against Terminal Drought in a Mediterranean-Type Climate

A Mediterranean-type climate with the frequent occurrence of terminal drought is typical of countries within Southern Europe, Middle Asia, South and Western Australia, North and South Africa, and some regions of California (United States), and Chile (Loss and Siddique, 1994; Acevedo et al., 1999). There are several reports confirming shifts in EFT/EM of 10–13 days in both bread and durum wheat cultivars over a century of breeding (Perry and D’Antuono, 1989; Araus et al., 2002; Álvaro et al., 2008; Isidro et al., 2011). These results were based on a comparison of growth between old and modern wheat cultivars in current environments, where the modern varieties consistently demonstrated an earlier start to flowering and more rapid life-cycle completion relative to older varieties. The authors concluded that a gradual rise in temperature and stronger terminal drought stimulated the selection of wheat genotypes with DE. The resultant modern varieties were significantly more productive due to minimization of the risk associated with drought stress during flowering and post-anthesis grain filling (Bidinger and Witcombe, 1989; Perry and D’Antuono, 1989; Acevedo et al., 1999; Álvaro et al., 2008; Araus et al., 2008; Hill and Li, 2016). The same trend for DE with EFT was predicted for the Australian ‘wheat belt’, where global warming is expected to result in an optimal FT 15 and 30 days earlier than current varieties by 2030 and 2050, respectively (Zheng B. et al., 2016). A subtler shift in flowering of around 2 days earlier is predicted for wheat in the growing regions of France (Gouache et al., 2012), however the development of EFT wheat cultivars with a DE mechanism could still prove to be a wise investment here, if global warming trends continue.

Current observations for wheat grown in Mediterranean-type climates have confirmed that wheat genotypes with a DE strategy show higher yield under terminal drought stress. In Cyprus, new successful durum wheat varieties (with higher yield) produced spikes 5–10 days earlier than the current standard, while a screen of barley lines showed a curvilinear correlation, where very early and very late flowering genotypes failed to match the yield of a local standard (Hadjichristodoulou, 1988). At ICARDA sites in Syria, 14 and 25 barley lines isolated from landraces (Acevedo and Ceccarelli, 1989; Van Oosterom and Acevedo, 1993), as well as 118 Doubled haploid lines from a barley mapping population, Nure × Tremois (Francia et al., 2011), and 13 lines of synthetic wheat (Inagaki et al., 2007), all showed an early heading time that significantly correlated with higher grain yield. Therefore, the increasing number of cases of non-EFT wheat genotypes overlapping with oncoming drought events triggered the general conclusion that “Yield under drought is often negatively correlated to anthesis date” (Acevedo and Ceccarelli, 1989).

In other regions with different climatic conditions involving heat and drought events, EF wheat genotypes with a DE strategy can also produce significantly higher yield. For example, five years of field trials in four South Asian countries (Bangladesh, India, Nepal, and Pakistan) showed a strong positive trend in high-yielding wheat breeding lines produced by CIMMYT and a negative correlation between yield and FT of tested wheat germplasms compared to local controls/checks (Mondal et al., 2013, 2016). In India and Pakistan, significantly higher grain yields in adapted EM wheat cultivars and mutant lines, respectively, were reported under conditions of terminal drought compared to non-EM controls (Nagarajan et al., 2008; Sial et al., 2008). These varieties were characterized as having a longer ‘time to heading’ and moderate grain filling duration, but with high grain filling rates enabling early maturation (Laxman et al., 2014). The exact opposite conclusion has been made based on a comparison of 75 old and modern Iranian bread wheat cultivars. With terminal drought, modern cultivars with highest grain yield showed EFT/EM, but grain filling time was longer compared to the old cultivars (Joudi et al., 2014). In the arid conditions of Saudi Arabia, where wheat is grown with surface drip irrigation, longer reproductive growth stages and grain filling periods were found to be more promising for high yield production in EM wheat genotypes under drought (Ihsan et al., 2016).

Anthesis and grain filling in wheat are the stages most sensitive to drought, when assimilates are mobilized from stem and leaves to seeds (Austin et al., 1977). Instead of an EFT/EM strategy, time of sowing can also be carefully optimized in order to escape drought at FT (Bodner et al., 2015). It was found in Northern Syria (Acevedo et al., 1999) and in Australia (French and Schultz, 1984; Brill, 2015) that late sowing significantly reduced grain yield in wheat due to the considerable overlap between the harsh drought/heat stress and the sensitive flowering stages. In contrast, early sowing increased grain yield in wheat, but only with mild to strong drought (Brill, 2015). Dry sowing is used in Western Australia, where seeds are sown in dry soil before the onset of the rain that marks the start of the wet season. This strategy allows escape from terminal drought when plants have EFT because seeds begin germination earlier, immediately after the first autumn rains (Fletcher et al., 2015). In both approaches, EFT occurs due to the earlier sowing time.

## Early Flowering Time and Early Maturing Wheat Can Have Comparatively High Yield Potential

Early flowering and maturity is an effective DE mechanism, but it can limit grain yield potential (Fischer, 1979; Turner, 1986; Bidinger and Witcombe, 1989) due to the reduced time available for photosynthetic production and seed nutrient accumulation necessary for higher grain yield (Ludlow, 1989; Radhika and Thind, 2014).

However, this is not always the case. Incidences where wheat with EFT/EM and SVP traits showed a HYP and produced more grain than wheat with traditional FT under non-stressed conditions have been described in both computer simulations and experimental approaches in field trails (Stapper and Fischer, 1990). For example, throughout Central Asia, EFT/EM wheat has been predicted to grow faster, produce more biomass and yield better in the face of climate change. A potential negative effect of EFT wheat with SVP in this region of Asia was not recorded in modeling, with possible limits on biomass accumulation found to be “more than counter-balanced by more favorable growth conditions” (Sommer et al., 2013). Experimentally, one such scenario was proven in ‘open-air facilities’ in Northern China and Tibet, where wheat plants grown in artificially warmed fields (0.5–1°C higher than that of non-warmed fields) produced more green leaf area, spikes and biomass, and finally increased yield by 8.4–11.4%, in association with both EFT and SVP of 5–9 and 13–14 days, respectively (Zheng et al., 2015; Zheng C. et al., 2016).

There are published reports describing EFT/EM wheat germplasms producing significantly improved HYP in the absence of drought. A study involving the introgression of *Vrn-B1* alleles into breeding material with different genetic backgrounds showed a change to EFT by an average of 2.6 days and a significantly increased HYP in non-stressed conditions in field trials, by the main yield components of both grain per spike and spike weight (Iqbal et al., 2007; Nitcher et al., 2014).

In Western Australia, two commercial EM wheat cultivars and five early/very early-maturing breeding lines produced significantly higher grain yield compared to commercial later-maturating wheat controls in two sites over four seasons (Regan et al., 1997). The analysis of yield components indicated a higher Harvest Index (better partitioning of dry matter in grains), and fewer spikelets but with higher seed weight in EM wheats, which were significant in value compared to controls. Because the vegetative period was shorter in EM wheats, the plants had better utilization of solar energy, nutrients and water, resulting in more rapid growth. The presented results indicated that WUE was significantly higher among all studied EM cultivars and very early maturing breeding lines compared to controls (Regan et al., 1997).

Commercial cultivars of wheat with EFT/EM traits must have an exceptional HYP. This depends on the combination of strong and vigorous early growth, high nutrient and WUE, balanced photosynthesis and respiration, and the production of more biomass prior to anthesis for fast and effective uptake of metabolites into seeds, all culminating in high yield and good quality wheat grain (Slafer et al., 1994; Richards, 1996; Passioura, 2012). Without HYP, the traits of EFT/EM have a positive effect on commercial wheat production only when limited by terminal drought conditions.

## Practical Perspectives

Drought escape is an important strategy that can improve crop yields. It is beneficial for yield stability of crops such as wheat under conditions of an approaching terminal drought. In this scenario, EFT and SVP are beneficial relative to plants with a later FT and longer vegetative period. In favorable conditions, if no drought event occurs, the advantages of EFT/EM can be detrimental to yield. However, there are several cases indicating that the boost seen in the growth of EFT/EM wheat plants can produce significantly higher grain yield potential even under non-stressed conditions.

Ideally, wheat farmers should be able to choose which varieties to plant based on the relative probability of a future terminal drought and balance their risk by using cultivars with the FT and vegetative phase that best suits their conditions. Mismatches in the prognosis and choice of wheat genotypes can, of course, result in losses at harvest.

Biologically, a DE growth habit does not mean that wheat plants with EFT/EM traits are more or less sensitive to drought. Indeed, the DE strategy assumes active growth and metabolism for the rapid completion of the life-cycle before drought events occur. However, if drought stress occurs earlier DE plants can gradually switch to DA with the succulent strategy or a more advanced drought tolerance strategy such as osmolyte production and high WUE (Ashraf et al., 2011). This can provide flexibility and a greater adaptive capacity in wheat plants exposed to the changed environments (Zlatev and Lidon, 2012). There is no biological barrier to producing or finding recombinant breeding lines from hybrids combining genes for both EFT and tolerance to dehydration/drought (Fitter and Hay, 2002). To illustrate this point, wheat plants show a range of plasticity in response to terminal drought. The development of both shallow and deep roots in wheat plants is reported to be advantageous for grain yield in conditions of either early or late terminal drought (Ehdaie et al., 2012). Another example of the combined plant strategy and plasticity can be seen in the newly developed EM bread wheat cultivar Konde INIA in southern Chile. It was reported that this variety produced 20% higher yield potential in non-stressed conditions compared to controls in addition to being well adapted to drought stress (Jobet et al., 2015). Similar results were reported in sorghum (Reddy et al., 2009), and in winter wheat in Hungary, where EM cultivars showed the best yield average overall after three years of field trials with ideal conditions, and moderate and severe drought, compared to varieties with a regular maturing time (Ágoston and Pepó, 2006). In legumes, a single EFT/EM groundnut cultivar Fleur 11 was reported as the most productive one among four studied, both in well-watered and drought field trials, where an efficient DE strategy was associated with HYP, quick growth and high WUE (Clavel et al., 2005).

A breeding program has been established based on similar wheat genotypes, with the potential to support grain yield both in the absence and presence of early drought at vegetative and pre-anthesis stages (Bidinger and Witcombe, 1989) but greater effort is required in this research and breeding area. Farmers should be able to choose from a wide array of genotypes with a pattern of maturity suitable for their given location and sowing time (Zheng et al., 2013). Wheat cultivars with EFT/EM traits can provide high yield in both terminal drought and non-stressed conditions.

## Author Contributions

YS generated an idea and wrote first draft of the manuscript. LZ and AK collected data for section 1 and edited all sections after completing. SJ and VS collected data, wrote, and revised the Sections “EFT and EM in Wheat Represents Successful Strategies Against Terminal Drought in a Mediterranean-Type Climate” and “EFT and EM Wheat Can Have Comparatively HYP”. FK and SdG collected data and completed the Sections “Mechanism of DE in Plants: Early Flowering” and “DE and EFT/EM Can Provide Benefits to Plants Under Drought Stress”. KS edited the Section “Comparison of DE and DA”. PL coordinated the all parts of the review and made final corrections and revisions.

## Conflict of Interest Statement

The authors declare that the research was conducted in the absence of any commercial or financial relationships that could be construed as a potential conflict of interest.

## Abbreviations

DA: drought avoidance
DE: drought escape
EFT: early flowering time
EM: early maturing
FT: flowering time
HYP high yield potential SVP: short vegetative phase
WUE: water use efficiency.
